# Exploring Cerebrovascular Function in Osteoarthritis: “Heads‐up”

**DOI:** 10.14814/phy2.14212

**Published:** 2019-10-29

**Authors:** Baraa K. Al‐Khazraji, Mark B. Badrov, Mason Kadem, Navena R. Lingum, Trevor B. Birmingham, Joel Kevin Shoemaker

**Affiliations:** ^1^ School of Kinesiology, Faculty of Health Sciences Western University London Ontario Canada; ^2^ Brain and Mind Institute Western University London Ontario Canada; ^3^ School of Physical Therapy Faculty of Health Sciences Western Ontario London, Ontario Canada; ^4^ Bone and Joint Institute Western University London Ontario Canada; ^5^ Department of Physiology and Pharmacology, Schulich School of Medicine and Dentistry Western University London Ontario Canada

**Keywords:** Cerebrovascular control, magnetic resonance imaging, osteoarthritis, transcranial Doppler ultrasound, white matter lesions, cardiovascular disease risk factors

## Abstract

Individuals with osteoarthritis (OA) are at greater risk of cardiovascular and cerebrovascular incidents; yet, cerebrovascular control remains uncharacterized. Our primary outcome was to acquire cerebrovascular control metrics in patients with OA and compare measures to healthy control adults (CTL) without OA or cardiovascular complications. Our primary covariate was a 10‐year risk factor for cardiovascular and stroke incidents, and secondary covariates were other cardiovascular disease risk factors (i.e., body mass index, carotid intima media thickness, and brachial flow‐mediated dilation). Our secondary outcomes were to assess anatomical and functional changes that may be related to cerebrovascular reactivity were also acquired such as white matter lesion volume and brief cognitive assessments. In 25 adults (*n* = 13 CTL, *n* = 12 OA), under hypercapnia, magnetic resonance imaging (3T) was used to acquire a “Global Cerebrovascular Reactivity” index across the larger intracranial cerebral arteries and white matter lesions, and transcranial Doppler was used for both middle cerebral artery hemodynamic responses to hypercapnia and to assess autoregulation via a sit‐to‐stand task. Compared to CTL, OA had lower “Global Cerebrovascular Reactivity” index responses to hypercapnia, autoregulatory responses, and greater white matter lesions (*P* < 0.05). These differences persisted after covarying for the outlined primary and secondary covariates. Patients with OA, in the absence of known cardiovascular disease, can exhibit pre‐clinical and impaired (compared to CTL) peripheral and cerebrovascular control metrics.

## Introduction

All‐cause mortality in people with osteoarthritis (OA) is related to cardiovascular disease (CVD) (Calvet et al. 2016; Hall et al. 2016; Veronese et al. 2016), and a greater risk of cerebrovascular disease such as stroke (Hsu et al. 2017). Osteoarthritis shares several disease characteristics with CVD and cerebrovascular disease: hypertension (Veronese et al. 2017), arterial intima media thickening (IMT) (Hoeven et al. 2013), and endothelial dysfunction (via flow‐mediated dilation; FMD) (Alkatan et al. 2016). Osteoarthritis often includes chronic low‐grade inflammation, both locally at the involved joint(s) and systemically (summarized in a recent review (Al‐Khazraji et al. 2018)). Importantly, systemic inflammation negatively impacts cerebrovascular health via disrupted blood–brain barrier (Kuhlmann et al. 2009; Hsuchou et al. 2012), cerebral small vessel disease (Pantoni, 2010), and stroke (Rost et al. 2001; Patgiri et al. 2014; Yu et al. 2017). Such cerebrovascular dysfunctions can, in part, lead to long‐term neural or functional damage as seen with cerebral microvascular damage indicated by white matter lesions and/or play a role in cognitive impairment (McKetton et al. 2019). Causal studies are unavailable, yet prevalence of dementia remains greater in people with OA compared to those without OA (Weber et al. 2019). This includes vascular‐related dementia, whose underlying neuropathology is caused by dysfunctional cerebrovasculature (Raz et al. 2016). Despite shared risk factors with cerebrovascular disease, presence of chronic inflammation, and potential risk of cerebrovascular impairment, cerebrovascular physiology remains unstudied in OA.

Cerebrovascular function generally is characterized by: cerebrovascular reactivity (to vasoreactive stimuli such as carbon dioxide; CO_2_) and cerebral autoregulation (cerebral blood flow maintenance in the face of systemic blood pressure fluctuations). Cerebrovascular reactivity is impaired in patients with acute ischemic stroke (Salinet et al. 2015), and in patients with cerebral small vessel disease suffering from lacunar strokes (Deplanque et al. 2013). Autoregulation indices predicted Glasgow Outcome Scale in a sample of patients after traumatic brain injury (Rivera‐Lara et al. 2017), impaired autoregulation is associated with brain atrophy and worse long‐term functional status in older adults with ischemic stroke (Aoi et al. 2012). Although cerebrovascular disease risk factors are expressed by patients with OA, there is a paucity of research that quantifies cerebrovascular control in such patients.

Therefore, the purpose of this study was to use ultrasound and magnetic resonance imaging (MRI) modalities to explore cerebrovascular control in individuals with knee OA, yet without known cardiovascular/cerebrovascular disease. This study was exploratory in nature and is the first to assess cerebrovascular physiology in a convenience sample of individuals with OA who otherwise have not experienced vascular‐related incidences.

## Methods

### Overall study design

For our primary objective, we combined hemodynamic, ultrasound imaging, and magnetic resonance imaging data to assess cerebrovascular control, operationally defined by: (1) middle cerebral artery (MCA) flow reactivity in response to hypercapnia (elevated levels of end‐tidal CO_2_), (2) an index of averaged cerebrovascular reactivity in larger intracranial arteries, referred to as “Global Cerebrovascular Reactivity”, and (3) an index of MCA dynamic cerebral autoregulation in response to a step‐wise reduction in mean arterial pressure (i.e., sitting to standing positions), referred to as Rate of Regulation (RoR). We focused on hemodynamics, flow reactivity and autoregulation in the MCA as it is easily accessible and is the most common site for cerebral infarction (Hedna et al. 2013). We then assessed the meta‐variables that were used to calculate “Global Cerebrovascular Reactivity,” which included comparing reactivity of nine intracranial vessels between the two groups.

In cardiac patients that exhibit several of the CVD risk factors that are generally found in people living with OA (Hoeven et al. 2013; Alkatan et al. 2016; Veronese et al. 2017), cerebrovascular reactivity and global cognitive scores are reduced in brain regions associated with cognition (Anazodo et al. 2015). For our secondary objective, we explored: (1) between‐group brain anatomical differences such as whole brain, gray and white matter volumes as blood flow changes may accommodate brain volumes, (2) early signs of small vessel damage indicated by white matter lesions, and (3) modest insight on cognitive capacity, via brief cognitive assessments, as there may be a link between cerebrovascular health and cognition.

Cerebrovascular outcomes related to OA are difficult to study independent of other co‐existing conditions; as such, primary analyses included comparing cerebrovascular function between those with and without OA while covarying for the 10‐year risk rate for CVD and stroke (Kim et al. 2016). The 10‐year risk rate for CVD and stroke is calculated by processing an individual’s various identified risk factors (e.g., age, sex, cholesterol, high‐density lipoprotein, systolic blood pressure (SBP), smoking history, diabetes mellitus) into a single composite value where a greater value indicates greater risk of CVD or stroke onset. Our secondary covariates included other risk factors which are not used to calculate the 10‐year risk score and were included as independent covariates in our analyses: body mass index (BMI), IMT, and FMD. The SBP was also accounted as an independent covariate as a means of sensitivity analysis, despite being included in the 10‐year risk score, as it is a predominant risk for white matter lesions and other cerebrovascular pathology.

For our secondary analyses, we compared the meta‐variables (i.e., individual vascular reactivity across nine intracranial vessels) that make up the “Global Cerebrovascular Reactivity” variable, between individuals with and without OA while covarying for our primary covariate. We also looked at between‐group differences of white matter lesions and brief cognitive assessments while accounting for the primary covariate.

### Eligibility criteria

We recruited a convenience sample of 12 patients previously diagnosed with knee OA, who had no known history of CVD, were 40–75 years of age and were not using anti‐inflammatory medication. All patients had symptomatic and radiographic knee OA confirmed by their primary care physician (Altman et al. 1986). We also recruited 13 healthy controls (CTL) with no knee symptoms and no known history of arthritis (e.g., osteoarthritis, rheumatoid, gout, etc.), or CVD. Participants were excluded if they were smokers, pregnant, or had any of the following conditions: Raynaud’s disease, respiratory illnesses, diabetes, claustrophobia, history of psychosis, eating disorders, manic or bipolar disorder, major psychiatric conditions, or dependence on alcohol or drugs. All participants provided written informed consent and were excluded if they were unable to provide written informed consent, or complete forms due to language or cognitive difficulties. The Human Subjects Research Ethics Board at the University of Western Ontario (London, Ontario, Canada) approved the experiment protocols herein.

### Anthropometrics, blood work, cognitive tests, and risk scores

Testing occurred over two sessions (each completed by 12 pm) separated by one to 30 days that involved a laboratory session and a neuroimaging session. All participants refrained from exercise, alcohol, non‐steroidal anti‐inflammatory drugs, and caffeine 12 h prior to all testing, and fasted 8 h for the blood sample.

Age, height, and weight were collected. The fasted blood plasma was analyzed for lipids, glucose, insulin, high‐sensitivity C‐reactive protein (hs‐CRP), and glycated hemoglobin (HbA1c) levels. Participants completed a health history form and two cognitive assessments: (1) the Montreal Cognitive Assessment (MoCA), a screening test for mild cognitive impairment (Nasreddine et al. 2005), and (2) the Trail Making Test (TMT) A and B, a neuropsychological assessment of speed of processing, mental flexibility, and executive functioning (Tombaugh 2004).

### Hemodynamics and cerebrovascular reactivity of the MCA

While supine, 5 min baseline data were collected which included continuous beat‐by‐beat mean arterial pressure (MAP), heart rate, end‐tidal partial pressure of carbon dioxide (PETCO_2_), and MCA blood velocity. After 5 min of breathing room air, participants breathed a gas mixture with elevated CO_2_ levels (5% CO_2_, 95% O_2_) using a 5L Douglas bag and a ventilation tube attached to the bottom of the face mask, and then 3 min of recovery while breathing room air. Using transcranial Doppler, the MCA was insonated with a 2‐MHz ultrasound probe placed at the temporal window and peak blood flow velocity envelope was collected using the Neurovision TCD System (Multigon Industries Inc., NY). Continuous beat‐to‐beat arterial blood pressure was monitored using a Finapres^®^ Finometer system, where a finger cuff was placed on the middle phalange of the third finger, and the finger blood pressure was calibrated with an upper arm cuff (Finapres^®^ Medical Systems, Amsterdam, Netherlands).To assess pressure‐independent changes in MCA velocity, a surrogate for vascular conductance was measured as the quotient between MCA velocity and corresponding mean arterial pressure (MAP). All devices were connected to a PowerLab data acquisition system (ADInstruments, Dunedin, Otago, New Zealand) and saved for offline analysis using LabChart software (LabChart Pro v.8, ADInstruments, Dunedin, Otago, New Zealand).

### Cerebrovascular autoregulation in MCA using a sit‐to‐stand protocol

The sit‐to‐stand protocol elicits a rapid but transient reduction in systemic blood pressure leading to concurrent changes in MCA blood flow, enabling the opportunity to study compensatory myogenic cerebrovascular responses (Sorond et al. 2009). To begin, participants sat upright, and without a back support. After 3 min of baseline data acquisition, participants stood unassisted for 3 min before resuming the seated position. Three sit‐to‐stand tasks were completed, and a minimum 10 mmHg drop in mean arterial pressure MAP was considered acceptable. Rate of regulation (RoR) was calculated using the method described by Aaslid et al. (1989) and Labrecque et al. (2019), described in brief as calculating the ratio between the normalized slope of cerebrovascular resistance (to the MCA blood velocity nadir time point) to the corresponding normalized drop in mean arterial pressure (Aaslid et al. 1989).

### Magnetic resonance imaging (MRI) of cerebrovascular responses and brain anatomy

All imaging was conducted on a 3.0 T MRI (Prisma Fit, Siemens, Erlangen, Germany) using a 32‐channel head coil. An MRI‐compatible respiratory plethysmography belt was used for breathing rate tracking, and a face mask for PETCO_2_ measurement. Imaging details are below.

#### Large cerebral artery reactivity

A T1‐weighted anatomical scan was obtained prior to, and during a hypercapnic stimulus (5% CO_2_, 95% O_2_). The hypercapnia image was initiated 2 min following initiation of the hypercapnia to capture steady state conditions (Coverdale et al. 2014). The T1‐weighted 3D SPACE pulse sequence used a radial trajectory, producing black‐blood with little flow artifact, improving signal contrast between vessel lumen and tissue. High‐resolution images (0.72 mm isotropic, whole brain) were acquired in the sagittal orientation (TE = 11.0 msec, TR = 700 msec, FOV = 230 mm, BW = 625Hz/px, iPat = 3, TA = 5:28 min).

#### Primary covariate: 10‐year risk rate for CVD/stroke index

The 10‐year risk of heart disease or stroke index was calculated using the algorithm presented by American College of Cardiology/American Heart Association Task Force (Goff et al. 2013). For example, the 10‐year risk for 55‐year‐old, nonsmoker without diabetes, a total cholesterol level of 213 mg/dL, high‐density lipoprotein cholesterol level of 50 mg/dL, and untreated systolic BP of 120 mmHg will result in a predicted risk factor value of 2.1% for white women, and 5.3% for white men (Goff et al. 2013).

#### Secondary covariates: BMI, IMT, FMD, and SBP

The BMI and SBP were collected anthropometric and baseline hemodynamic recordings.

Right carotid artery IMT was measured using B‐mode ultrasound imaging (GE Vivid 7, 10 MHz), and all diameters and IMT were measured offline using EchoPAC software (GE Healthcar).

The FMD protocol was conducted in the right brachial artery. While supine, the right brachial artery was imaged using duplex ultrasonography (GE Vivid 7, 10 MHz imaging transducer; 4.7 MHz Doppler ultrasound imaging). A 5‐inch wide Hokanson cuff was placed 5 cm distal to the antecubital fossa and attached to a Hokanson rapid cuff inflator (E20 Rapid Cuff Inflator, Hokanson, D. E. Hokanson Inc., Bellevue, WA). The ultrasound probe position was fixed to ensure stable imaging at an insonation angle of 60°. The FMD protocol included a 1‐min baseline (brachial diameter images captured within 1 min before inflation), followed by a 5‐min cuff inflation (200 mmHg), and 3 min of recovery. Brachial artery diameter images were captured at 15, 30, 45, 50, 55, 60 sec of the first minute, 5, 10, 15, 30, 45, 60 sec in the second minute, and at 15, 30, 45, and 60 sec in the third minute (Coverdale et al. 2017). Continuous spectral mean flow velocity signal was measured throughout.

#### Whole brain volume

A 3DT1 scan was performed for full brain volume analysis. The following imaging parameters were used: TE = 2.98 msec, TR = 2300 msec, FOV = 256 mm, BW = 240 Hz/px, iPat = 2, TA = 5:21 min.

#### White matter hyperintensities

A fluid‐attenuated inversion recovery (FLAIR) scan was acquired for analysis of white matter hyperintensity volumes (TE = 387 msec, TR = 5000 msec, FOV = 230 mm, BW = 751 Hz/px, iPat = 2, TA = 5:42 min).

### MRI analysis

Cross‐sectional area (CSA) of the basal cerebral conduit vessels were measured from T1‐weighted scans using multiplanar reconstruction in OsiriX software (Pixmeo^©^, Bernex, Switzerland). Measures included bilateral (L‐left, R‐right) subcranial internal carotid (LICA and RICA) arteries, and the major arteries in the circle of Willis including the basilar (BA), bilateral posterior cerebral (LPCA and RPCA), middle cerebral (LMCA and RMCA), and anterior cerebral (LACA and RACA) arteries during baseline and elevated CO_2_ T1 scans. In the coronal plane, cross‐hairs were positioned parallel to the longitudinal slice of the artery, and a blinded trained observer measured CSAs. Cerebrovascular reactivity across the basal conduit arteries was measured as the percent change in CSA divided by the change in PETCO_2_ during hypercapnia. Similarly, the MCA cerebrovascular flow reactivity (%/mmHg) was measured as percent change in calculated flow (from baseline) divided by the change in PETCO_2_ during hypercapnia. To provide an overall characteristic of cerebrovascular reactivity between groups, “Global Cerebrovascular Reactivity” index was calculated as the average of cross‐sectional reactivity responses across the nine intracranial vessels.

Brain images were evaluated for white and gray matter volume, and white matter hyperintensities were assessed for measures of white matter lesions. Whole brain volume (without ventricles), white and gray matter volume, and white matter hyperintensity lesions were obtained from 3DT1 images. Brain volumes were determined using the FreeSurfer software, version 5.3.0 (http://surfer.-nmr.mgh.harvard.edu/).

Based on Schmidt et al*.* (2012), white matter hyperintensity lesions were segmented using the lesion growth algorithm from the Lesion Segmentation Toolbox (www.statistical-modelling.de/lst.html) for Statistical Parametric Mapping software (SPM12, http://fil.ion.ucl.ac.uk/spm). A pre‐determined initial threshold (*κ* = 0.30) was used to obtain binary lesion maps. Lesion volumes (mm^3^) were summed within an individual and reported as group averages.

### Statistical analyses

All statistical analyses were conducted using IBM^®^ SPSS^®^ Statistics software (v.25; IBM Corp., Armonk, NY). Univariate general linear model (GLM) analyses were conducted to assess between‐group differences for all measured and calculated experimental variables. Primary or secondary covariate analyses were conducted depending on the variable of interest: (1) cerebrovascular control metrics were assessed between groups while covarying for primary and secondary covariates, (2) between‐group individual vessel reactivity across the nine intracranial large cerebral arteries that make up the Global Cerebrovascular Reactivity index were analyzed using GLM and the primary covariate, and (3) brain volumes, white matter lesions, and cognitive assessments were compared using a GLM; and white matter lesions and cognitive assessments were covaried for the primary covariate but also for SBP as it is a strong factor in development of lesions. Where differences existed, cognitive assessments were covaried for white matter lesion volume. An alpha‐level of *P* < 0.05 was selected, and effect sizes are reported as eta squared values (*η* ^2^), where a large effect size was accepted when *η* ^2^ > 0.14. Where possible, between‐group mean differences and 95% confidence intervals were calculated, with unadjusted and adjusted means provided in tables.

## Results

### Participant characteristics, blood analysis, and hemodynamics

The CTL and OA groups were of similar age. The BMI and SBP measurements were greater in OA, and diastolic blood pressures were similar between groups (Table 1).

**Table 1:** Participant characteristics, blood analysis, and hemodynamic measurements during hypercapnia.

	CTL	OA	MD (95% CI)	*P*‐value	*η* ^2^	PETCO_2_ and Hemodynamics	CTL	OA	MD (95% CI)	*P*‐value	*η* ^2^
Characteristics											
*n*; males/females	13; 6/7	12; 6/6				BL PETCO_2_	45 ± 5	43 ± 6	2 (−3,6)	0.48	0.026
Age (y)	57 ± 12	64 ± 7	−7 (−15,1)	0.10	0.11	HC PETCO_2_	47 ± 6	48 ± 5	−1 (−7,4)	0.58	0.016
BMI (kg/m^2^)	26 ± 2	30 ± 6	−5 (−8,−1)	**0.02**	0.22	BL MAP	91 ± 11	96 ± 15	−5 (−17,6)	0.34	0.044
Seated SBP (mmHg)	126 ± 11	139 ± 12	−12 (−22,−3)	**0.01**	0.24	HC MAP	95 ± 11	104 ± 17	−8 (−21,4)	0.19	0.081
Seated DBP (mmHg)	79 ± 6	80 ± 9	−1 (−9,6)	0.72	0.006	BL Velocity	58 ± 8	54 ± 8	4 (−4,13)	0.30	0.051
Blood analysis						HC Velocity	72 ± 12	63 ± 15	9 (−3,20)	0.13	0.11
Glucose (mmol/L)	5.4 ± 0.6	5.3 ± 0.6	0.05 (−0.4,0.5)	0.83	0.002	BL Conductance	0.65 ± 0.11	0.58 ± 0.18	0.07 (−0.05,0.2)	0.25	0.064
Cholesterol (mmol/L)	4.6 ± 1.1	5.3 ± 1.1	−0.7 (−1.6,0.2)	0.12	0.10	HC Conductance	0.76 ± 0.11	0.63 ± 0.20	0.12 (−0.02,0.3)	0.09	0.135
HDL (mmol/L)	1.6 ± 0.3	1.4 ± 0.3	0.2 (−0.05,0.4)	0.12	0.10						
LDL (mmol/L)	2.7 ± 0.6	3.3 ± 0.9	−0.6 (−1.2,0.06)	0.08	0.13						
Triglycerides (mmol/L)	1.0 ± 0.3	1.3 ± 0.4	−0.3 (−0.6,−0.04)	**0.03**	0.19						
HbA1C (%)	5.4 ± 0.4	5.4 ± 0.4	−0.06 (−0.4,0.2)	0.70	0.006						
hsCRP (mg/mL)	1.4 ± 1.3	2.5 ± 2.4	−1.1 (−2.8,0.6)	0.18	0.08						
Insulin (pmol/L)	50 ± 41	72 ± 43	−21 (−56,13)	0.22	0.07						

Univariate general linear model (GLM) comparing values between control (CTL), and those with osteoarthritis (OA). Between‐group mean differences (MD) with 95% confidence intervals (CI; upper, lower bounds) and values in mean ± SD are provided. MAP and PETCO_2_ are in mmHg, MCA conductance in cm/s/mmHg, and MCA velocity in cm/s. BL, baseline; HC, hypercapnia. Bolded *P*‐values indicate significance (*P* < 0.05).

Glucose, cholesterol, high‐density lipoprotein (HDL), low‐density lipoprotein (LDL), HBA1C, high‐sensitivity C‐reactive protein (hsCRP), and insulin were similar across groups. Compared to the CTL group, those with OA had higher triglycerides (Table 1).

The PETCO_2_, MAP, MCA blood velocities, and MCA vascular conductance were similar between groups at baseline and during hypercapnia (Table 1).

### Primary and secondary covariates

The 10‐year risk score for CVD/stroke was greater in those OA than in CTL (Table 2). Carotid IMT was greater in OA than in CTL, while brachial artery FMD was lower in OA than in CTL (Table 2).

**Table 2:** Primary and secondary covariates used in primary and secondary analyses of cerebrovascular control variables.

	Covariate Number	CTL	OA	MD (95% CI)	*P*‐value	*η* ^2^
Primary covariate						
10‐year risk factor for CVD/stroke	1	6 ± 6 (*n* = 13)	11 ± 5 (*n* = 12)	−5 (−10, −1)	**0.03**	0.18
Secondary covariates						
BMI (kg/m^2^)	2	26 ± 2 (*n* = 13)	30 ± 6 (*n* = 12)	−5 (−8, −1)	**0.02**	0.22
FMD (%)	3	9.5 ± 4.3 (*n* = 13)	3.7 ± 2.5 (*n* = 10)	5.9 (2.7,9.0)	**0.001**	0.41
IMT (mm)	4	0.43 ± 0.05 (*n* = 13)	0.55 ± 0.07 (*n* = 10)	−0.13 (−0.18, −0.08)	**<0.001**	0.58
SBP (mmHg)	5	126 ± 11 (*n* = 13)	139 ± 12 (*n* = 12)	−12 (−22, −3)	**0.01**	0.24

Univariate GLM comparing between‐group primary and secondary covariates used in subsequent statistical analyses in CTL and OA: body mass index (BMI), brachial artery flow‐mediated dilation (FMD), carotid artery intima‐media thickness (IMT), systolic blood pressure (SBP). Between‐group mean differences (MD) with 95% confidence intervals (CI; upper, lower bounds) and values in mean ± SD are provided. Bolded *P*‐values indicate significance (*P* < 0.05).

### Cerebrovascular control: MCA blood flow reactivity, global cerebrovascular reactivity, and autoregulation

**Figure 1:**
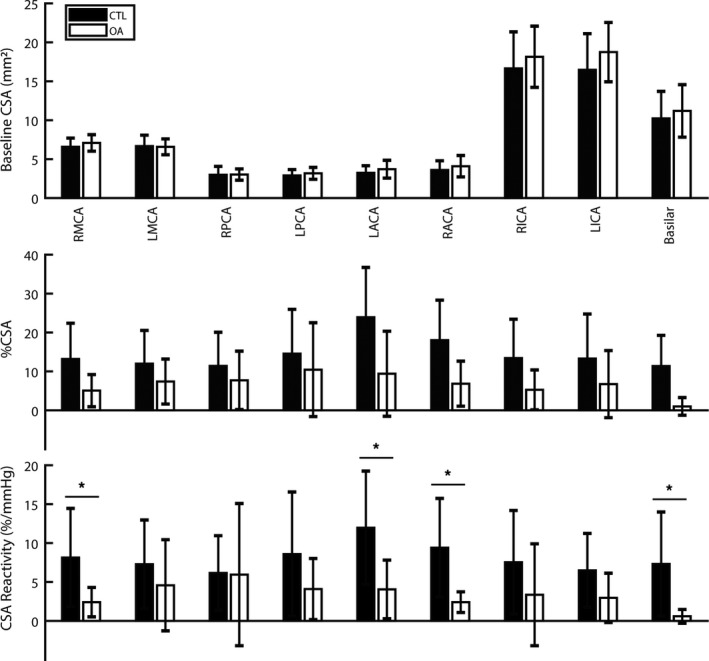
Unadjusted baseline cross‐sectional area (CSA), percent change from baseline in response to hypercapnia (%CSA), and reactivity (CSA Reactivity; %/mmHg) are shown for the bilateral (right, R; left; L) middle (MCA), posterior (PCA), anterior (ACA) cerebral arteries, and the internal carotid (ICA) and basilar intracranial arteries across control (CTL) and osteoarthritis (OA) individuals. Values presented as mean ± S.D. Between‐group raw mean differences are indicated with * to indicate OA different from CTL after covarying for the primary covariate of 10‐year risk factor for CVD/stroke.

Between groups, baseline cross‐sectional areas were similar across the nine intracranial large cerebral arteries (Fig. 1). Individual vessel reactivities were different between the groups; specifically, RMCA, LACA, RACA, and basilar CSA reactivity were lower in the OA group compared with CTL (Fig. 1).

**Figure 2:**
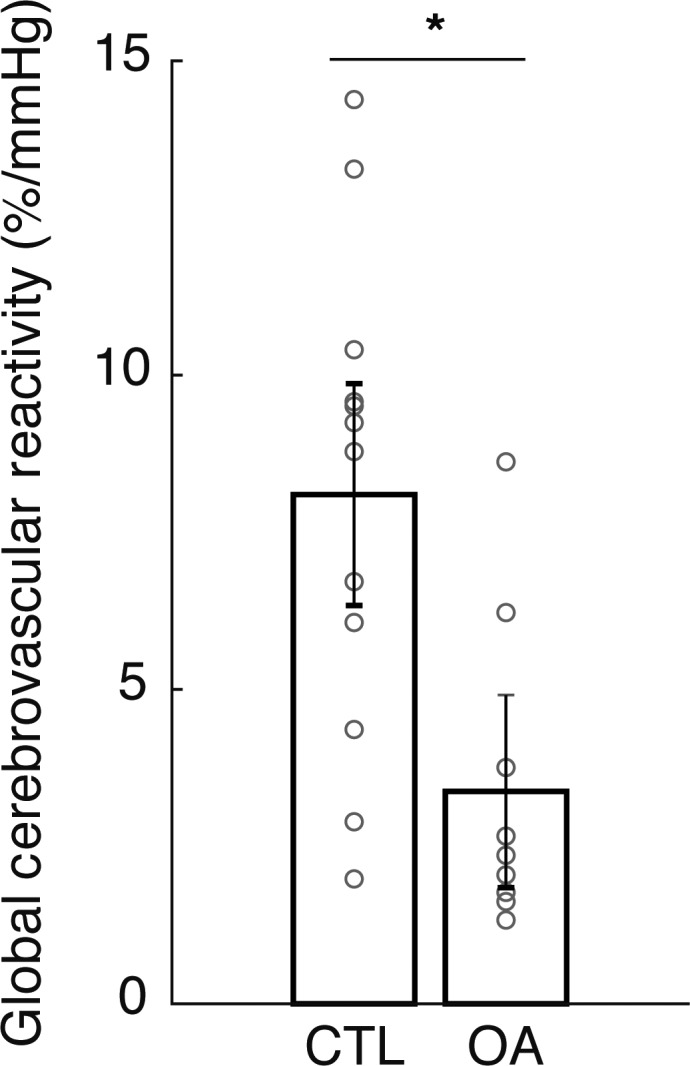
Unadjusted between‐group (control, CTL; osteoarthritis, OA) cross‐sectional area reactivity (from baseline) during hypercapnia averaged across the nine larger intracranial cerebral arteries. Both individual participant data (circles) and mean ± S.D. are shown. Between‐group raw mean differences are indicated with * to indicate OA different from CTL, with specific between‐group differences after primary and secondary covariate analyses detailed in Table 2.

The resting (pre‐CO_2_) MCA calculated blood flows were 225 ± 50 mL/min versus 233 ± 69 mL/min (CTL vs. OA; mean ± SD) with a *P*‐value of 0.78, indicating baseline MCA calculated blood flow values were similar between groups. The MCA Blood Flow Reactivity, Global Cerebrovascular Reactivity (averaged over 9 vessels/individual) and RoR were greater in the CTL group than in the OA group even after accounting for primary and secondary covariates (Fig. 2 and Table 3).

**Table 3:** Between‐group comparisons of cerebrovascular control with and without covariates.

Covariate #	CTL	OA	MD (95% CI)	*P*‐value	*η* ^ 2^	CTL	OA	MD (95% CI)	*P*‐value	*η* ^ 2^	CTL	OA	MD (95% CI)	*P*‐value	*η* ^ 2^
	MCA Flow CVR					GCR					RoR				
	25 ± 5_u_ (*n* = 11)	7 ± 4_u_ (*n* = 9)	18 (4,32)_u_	**0.014**_u_	0.293	8.1 ± 1.1 (*n* = 12)	3.4 ± 0.8 (*n* = 9)	4.7 (1.6,7.8)_u_	**0.005**_u_	0.352	0.19 ± 0.01_u_ (*n* = 12)	0.11 ± 0.02_u_ (*n* = 10)	0.08 (0.04,0.1)_u_	**0.001**_u_	0.423
1	25 ± 5_u_	7 ± 4_u_	18 (2,34)_a_	**0.031**_a_	0.246	8.1 ± 1.1_u_	3.4 ± 0.8_u_	4.2 (0.8,7.6)_a_	**0.017**_a_	0.277	0.19 ± 0.01_u_	0.11 ± 0.02_u_	0.09 (0.04,0.1)	**0.002**_a_	0.419
	25 ± 5_a_ (*n* = 11)	7 ± 5_a_ (*n* = 9)				7.9 ± 1.0_a_ (*n* = 12)	3.7 ± 1.2_a_ (*n* = 9)				0.19 ± 0.02_a_ (*n* = 12)	0.10 ± 0.02_a_ (*n* = 10)			
2	25 ± 5_u_	7 ± 4_u_	14 (0,29)_a_	0.054_a_	0.202	8.1 ± 1.1_u_	3.4 ± 0.8_u_	5.2 (1.9,8.6)_a_	**0.004**_a_	0.372	0.19 ± 0.01_u_	0.11 ± 0.02_u_	0.09 (0.04,0.1)	**0.002**_a_	0.401
	24 ± 4_a_ (*n* = 11)	9 ± 5_a_ (*n* = 9)				8.3 ± 1.0_a_ (*n* = 12)	3.` ± 1.2_a_ (*n* = 9)				0.19 ± 0.02_a_ (*n* = 12)	0.10 ± 0.02_a_ (*n* = 10)			
3	25 ± 5_u_	5 ± 4_u_	23 (5,42)_a_	**0.015**_a_	0.332	8.1 ± 1.1_u_	3.6 ± 1.0_u_	2.9 (−1.2,6.9)_a_	0.148_a_	0.126	0.19 ± 0.01_u_	0.12 ± 0.02_u_	0.06 (0,0.1)	**0.039**_a_	0.228
	26 ± 5_a_ (*n* = 11)	3 ± 6_a_ (*n* = 7)				7.5 ± 1.0_a_ (*n* = 12)	4.6 ± 1.4_u_ (*n* = 7)				0.19 ± 0.02_a_ (*n* = 12)	0.13 ± 0.02_a_ (*n* = 8)			
4	25 ± 5_u_	10 ± 3_u_	25 (2,46)_a_	**0.032**_a_	0.256	8.1 ± 1.1_u_	3.0 ± 0.8_u_	6.4 (1.2,11.5)_a_	**0.016**_a_	0.295	0.19 ± 0.01_u_	0.10 ± 0.02_u_	0.11 (0,0.2)	**0.006**_a_	0.345
	29 ± 5_a_ (*n* = 11)	5 ± 7_a_ (*n* = 8)				8.6 ± 1.2_a_ (*n* = 12)	2.2 ± 1.6_a_ (*n* = 8)				0.20 ± 0.02_a_ (*n* = 12)	0.09 ± 0.02_a_ (*n* = 9)			
5	25 ± 5_u_	7 ± 4_u_	17 (1,33)_a_	**0.044**_a_	0.219	8.1 ± 1.1_u_	3.4 ± 0.8_u_	4.4 (0.8,7.9)_a_	**0.018**_a_	0.273	0.19 ± 0.01_u_	0.11 ± 0.02_u_	0.10 (0.1,0.2)	**<0.001**_a_	0.531
	25 ± 5_a_ (*n* = 11)	8 ± 5_a_ (*n* = 9)				7.9 ± 1.0_a_ (*n* = 12)	3.6 ± 1.2_a_ (*n* = 9)				0.20 ± 0.01_a_ (*n* = 12)	0.10 ± 0.02_a_ (*n* = 10)			

Middle cerebral artery flow cerebrovascular reactivity (MCA Flow CVR; %/mmHg), “Global Cerebrovascular Reactivity” (GCR; %/mmHg), and rate of regulation (RoR; s^‐1^) compared between control (CTL) and osteoarthritis (OA) using univariate GLM with covariates (where 1 ‐ 10‐year risk factor for CVD/stroke, 2 – BMI, 3 – FMD, 4 – IMT, and 5 – SBP). Values reported as mean ± SEM. Raw values are reported as mean ± SEM (indicated with subscript “u”) and corresponding adjusted mean ± SEM (indicated with subscript “a”), and between‐group mean differences (MD) with Bonferroni adjusted 95% confidence intervals (CI; upper, lower bounds) are provided. Bolded *P*‐values indicate statistically significant differences between groups (*P* < 0.05).

### Cerebral anatomy and cognitive assessments

Total brain volumes (without ventricles), and white and gray matter volumes were similar across groups, while white matter lesions were greater in OA than in CTL (Table 4). Compared to CTL, OA had lower MoCA scores, that remained different between the groups after primary covariate analysis but not after covarying for white matter lesion volumes (Table 4). The TMT A and B times were similar between groups (Table 4).

**Table 4:** Cerebral anatomical volumes, cognitive metrics, and index of cerebro‐microvascular damage.

	CTL	OA	MD (95% CI)	*P*‐value	*η* ^ 2^
MRI brain volumetric data					
Brain volume without ventricles (mL)	1067 ± 39 (*n* = 12)	1083 ± 41 (*n* = 9)	−16 (−131,98)	0.77_u_	0.005
Cortical gray matter volume (mL)	446 ± 14 (*n* = 12)	449 ± 17 (*n* = 9)	−3 (−49,43)	0.88 _u_	0.001
Cortical white matter volume (mL)	445 ± 17 (*n* = 12)	463 ± 20 (*n* = 9)	−18 (−74,38)	0.50 _u_	0.024
White matter lesions (mL)	0.29 ± 0.5 (*n* = 12)	2.9 ± 0.6 (*n* = 9)	−2.6 (−4.3, −0.9)_u_	**0.004**_u_	0.357
Covariate #1	0.29 ± 0.5_u_	2.9 ± 0.6_u_	−1.98 (−3.68, −0.29)_a_	**0.024_a_**	0.252
	0.55 ± 0.50_a_ (*n* = 12)	2.5 ± 0.6_a_ (*n* = 9)			
Covariate #5	0.66 ± 0.5_a_ (*n* = 12)	2.4 ± 0.6_a_ (*n* = 9)	−1.73 (−3.44, −0.03)_a_	**0.046_a_**	0.203
Cognitive assessments					
MoCA (score out of 30)	29 ± 1 (*n* = 13)	27 ± 2 (*n* = 11)	2 (1,3)_u_	**0.004_u_**	0.32
Covariate #1	29 ± 1_u_	27 ± 2_u_	2 (1,3)_a_	**0.012_a_**	0.267
	29 ± 1_a_ (*n* = 13)	27 ± 2_a_ (*n* = 11)			
Covariate: white matter lesions	29 ± 1_u_	27 ± 2_u_	1 (−1,3)_a_	0.32_a_	0.059
	29 ± 1_a_ (*n* = 12)	28 ± 1_a_ (*n* = 8)			
Trail making test A (seconds)	30 ± 2_u_ (*n* = 13)	29 ± 2_u_ (*n* = 11)	1 (−5,8)_u_	0.70_u_	0.007
Trail making test B (seconds)	54 ± 7_u_ (*n* = 13)	59 ± 7_u_ (*n* = 11)	−4 (−25,16)_u_	0.65_u_	0.01

Brain anatomy, white matter lesions, and cognitive assessments compared between control (CTL) and osteoarthritis (OA) using univariate GLM with primary and specific secondary covariate analyses (where 1 ‐ 10‐year risk factor for CVD/stroke, and 5 ‐ SBP) for white matter lesions and MoCA. Raw values are reported as mean ± SEM (indicated with subscript “u”) and corresponding adjusted mean ± SEM (indicated with subscript “a”), and between‐group mean differences (MD) with Bonferroni adjusted 95% confidence intervals (CI; upper, lower bounds) are provided. Bolded *P*‐values indicate statistically significant differences between groups (*P* < 0.05).

## Discussion

To the best of our knowledge, this is the first study to investigate cerebrovascular control in patients with OA. The main and summarized finding was that these patients expressed impairments in carbon dioxide‐induced dilatory and autoregulatory function compared to healthy controls, which persisted after statistically controlling for major co‐existing vascular disease risk factors. Therefore, these preliminary data point to an important and previously undetected cerebrovascular impairment in participants with knee OA. Importantly, these patients are not routinely screened for cerebrovascular impairments despite higher risk of for stroke. In these regards, the data provide scientific rationale for pursuing future work in this area of study.

The current group of participants with OA exhibited impaired peripheral vascular health, as well as dysfunctional cerebrovascular reactivity, autoregulatory capacity, and greater white matter lesion volumes, compared to healthy controls. Participants with OA were irregular consumers of medications, and otherwise undiagnosed with any vascular or neurological concerns. These data suggest an association between established OA and subclinical impairments in cerebrovascular control; however, we cannot conclude that our findings relate to or imply dysfunctions in cerebrovascular health. Our participants did not report symptoms of established cerebrovascular dysfunction (e.g., cerebrovascular disease, pre‐syncope symptoms). Whether our findings provide insight on future outcomes or progression of underlying subclinical impairments is not within the boundaries of this study. Such links require future studies that target longitudinal evaluation of cerebrovascular health outcomes in individuals with OA.

Abnormal peripheral vascular metrics, such as greater carotid intima media thickness (Hoeven et al. 2013) and decreased flow‐mediated dilation (Alkatan et al. 2016) are reported in OA. The current study supports these earlier reports. However, the current observations of progressive cerebrovascular dysfunction are novel in this patient group. “Global cerebrovascular reactivity” was calculated as an index for average cerebrovascular reactivity within the larger intracranial cerebral arteries. Since cerebrovascular reactivity was calculated as percent changes in cross‐sectional area during hypercapnia from baseline levels, we have accounted for inter‐vascular differences in baseline vascular dimensions. Therefore, this metric provides a global account for cerebrovascular reactivity and accommodates the anatomical and functional layout of the cerebrovascular network (Devault et al. 2008). Global reactivity was lower in the OA group, even after adjusting for the 10‐year CVD/stroke risk factor and other vascular disease risk factors. As a point of interest and further investigation, between‐group differences in individual vessel reactivity was present even after adjusting for the 10‐year CVD/stroke risk factor. These findings suggest that cerebrovascular impairment in the OA group occurred for reasons that are independent from common cardiovascular/cerebrovascular risk factors. In other words, these data suggest an independent impact of OA on cerebrovascular function.

Impaired cerebrovascular reactivity in both extracranial conduit vessel dilation (i.e., internal carotid arteries) and MCA blood flow indicate impaired dilatory responses to all levels of the MCA vascular bed. The impaired dilatory response to hypercapnia, as well as slowed autoregulatory responses to pressure‐dependent stimuli, suggest that dilatory impairments are a generalized feature of OA across multiple stimuli and cerebrovascular regions, although the mechanisms are unclear. In younger obese adults (mean age 37) with BMI > 40 kg/m^2^, vasomotor reactivity (to a 30‐sec apnea test) was lower than age‐matched non‐obese adults with BMI < 25 kg/m^2^ (Rodríguez‐Flores et al. 2014). However, in the current study, BMI did not account for the between‐group cerebrovascular reactivity (i.e., cerebrovascular cross‐sectional area) differences.

White matter lesions (WML) describe a range of neuronal damage, often related to microvascular damage (Launer 2004; Debette and Markus 2010). Therefore, an important observation of the present study was the high volume of WML in the participants with OA; however, these values are subclinical, as they reside outside of range volumes observed in patients with small vessel stroke (Rost et al. 2010) (reported as 3.4–14.7 cm^3^). Normally, periventricular white matter lesions are reported to be greater after total joint replacement (Jonsson et al. 2011), indicating an effect of OA disease progression on cerebral microvascular damage. While based on a limited number of participants, the current data provide the first evidence indicating subclinical cerebrovascular impairment occurring alongside abnormal (relative to CTL) WML volumes in patients with OA. Interestingly, these patients also exhibited lower scores on the cognitive assessments, and it has been suggested that WMHs have been linked with neurodegeneration thereby driving cognitive decline (Rizvi et al. 2017). Generally, hypertension is considered to be the primary contributor to the development of white matter hyperintensities, although conflicting evidence exists on whether this results in greater white matter hyperintensities occurrences across the whole brain, or specific to either periventricular or deep white matter areas of the brain (Moroni et al. 2018). In the current study, participants with OA had greater white matter hyperintensities even after adjusting for the 10‐year risk for CVD/stroke index or SBP (Table 4). Although, it is important to note that SBP seemed to have an important role in WML volumes as between‐group adjusted mean differences were lower than the unadjusted mean differences of WML volumes (Table 4).

Cardiovascular and cerebrovascular outcomes in osteoarthritis are documented, where individuals with osteoarthritis have a higher risk of experiencing a vascular‐related incident than those without osteoarthritis (Calvet et al. 2016; Hall et al. 2016; Hsu et al. 2017). In a population‐based cohort study examining all‐cause and disease specific mortality of adults with either knee or hip OA (>1000 individuals and >35 years of age), age‐ and sex‐standardized mortality and hazard ratios were higher in OA than those without OA, and of particular interest, excess mortality was pronounced for cardiovascular disease and dementia associated mortality (Nuesch et al. 2011). In the current study, the observed cerebrovascular‐related impairments in the presence of OA were present even after accounting for risk factors that may have impacted cerebrovascular control.

Although underlying mechanisms in OA were not the focus of the current study, potential mechanisms could include persistent low‐grade inflammation, metabolic disturbances, or factors related to limited physical activity due to OA symptoms. The comprehensive review by Zhuo et al. summarizes the inflammatory and oxidative stress mechanisms shared between OA and metabolic syndrome, and even suggest that metabolic OA should be considered as a sub‐feature of metabolic syndrome (Zhuo et al. 2012). In our previous narrative review, we provided a synthesis of literature focusing on cerebrovascular outcomes in osteoarthritis with a proposed shared underlying link of inflammation (Al‐Khazraji et al. 2018). There are overlapping inflammatory and oxidative mechanisms at the local and/or systemic level which may contribute to progression of OA and cerebrovascular dysfunction. Examples include the lectin‐like oxidized low‐density lipoprotein receptor‐1 (LOX‐1), interleukin‐6 (IL‐6), and reactive oxidative species (ROS). LOX‐1 is found on the vascular endothelium, and its activation via increased superoxide production can lead to vascular thickening and lipid deposition. Serum cholesterol and lipid deposition is greater within the joint during OA (Gkretsi et al. 2011), and a rodent study using a destabilization of the medial meniscus as a model (DMM) for OA concluded that LOX‐1 activation is required for OA progression (Hashimoto et al. 2016). Systemic plasma levels of IL‐6 are high in OA patients, and rodent studies using the DMM model for OA determined that IL‐6 activation is involved in catabolic pathways in the joint cartilage (Latourte et al. 2017). In a 7‐year long observational study conducted in participants with vascular risk factors, those with higher circulating levels of IL‐6 were more likely to develop dementia (Miwa et al. 2016). Finally, both cerebrovascular cells and chondrocytes have the ability to produce ROS. Higher levels of ROS in the cerebrovasculature can lead to compromised blood brain barrier integrity and a higher level of plasma NADPH oxidase activity in humans has shown to be positively correlated with greater cartilage degradation (Kim et al. 2015). Although these inflammatory mechanisms were not reported as simultaneous measures from the cerebrovasculature and the joint in an OA model, there are enough lines of evidence supporting systemic and local effects of shared inflammatory mechanisms that may contribute to concurrent degradation of cerebrovascular health and OA progression.

The results of this preliminary study indicate patients diagnosed with OA can express significant cerebrovascular dysregulation. Importantly, the impaired cerebrovascular reactivity to CO_2_ and pressure‐dependent stimuli are suggestive of vascular disease that exists at a subclinical state and would normally remain undetected in routine follow‐up clinical evaluations. As well, the observed cerebrovascular dysfunction is also accompanied by poorer performance on cognitive assessments. Of note, the MOCA scores, although lower in the group with OA, were in a normal range for this age (Nasreddine et al. 2005) but were explained by WML. Therefore, the current observations provide important insight into epidemiological studies that indicate high risk for OA patients for cardiovascular disease (Nuesch et al. 2011; Veronese et al. 2016), dementia (Nuesch et al. 2011), and cerebrovascular events (Hsu et al. 2017). How these events progress in OA participants remains unknown, but the covariate analyses suggest that these impairments are not mediated by known cardiovascular/cerebrovascular risk factors. The magnitude of cerebrovascular impairment points to a relationship between OA and cerebrovascular outcomes that may have critical implications later in life.

### Limitations

We relied on pre‐existing diagnoses of OA. We did not perform radiographic examinations or assess the extent of symptoms. We also did not perform radiographs on the CTL participants; thus, we cannot confirm participants did not have radiographic OA (despite self‐reported accounts of no knee symptoms and no OA history). Fully accounting for co‐morbid conditions in OA is difficult, particularly in an exploratory study with a limited number of participants. For example, we adjusted for the covariates independently to prevent overfitting (which may occur when accounting for numerous covariates (Babyak 2004)). Although our sample size was small, our effect sizes were considered large and the post‐hoc observed power (SPSS) ranged from 0.7 to 0.99 for our primary cerebrovascular control variables. Although we cannot discount the integrative role of comorbidities on cerebrovascular control, we attempted to adjust for the role of common and well‐established cardiovascular risk factors as well as a composite risk score. The 10‐year CVD/stroke risk factor is not without its limitations as more work is required in filling gaps on risk assessment and outcomes of different racial/ethnic groups across the age span (Goff et al. 2013). Furthermore, using specific, rather than composite, secondary covariates yielded differences between our groups. In this study, we attempted to include data from an equal number of males and females, and our participants were mostly Caucasian. Finally, the present sample of patients with OA is small and the extent of disease is unknown.

The reported vascular reactivity and autoregulation findings may differ depending on the context of the stimulus and analytical methods. Specifically, we reported vascular reactivity to hypercapnia; however, vasoreactivity to hypocapnia may produce different results than the current findings as reactivity differs between hypercapnia and hypocapnia in healthy humans (Coverdale et al. 2014; Al‐Khazraji et al. 2019). Our dynamic autoregulation findings in the MCA may not translate to other regions of the cerebrovasculature. One such study assessed whether orthostatic stress affected dynamic cerebral autoregulation by measuring rate of regulation (via bilateral thigh‐cuff release) at supine and during a 60‐degree head‐up tilt maneuver. Although supine dynamic cerebral autoregulation was similar, there was a greater attenuation in the dynamic cerebral autoregulation response of the vertebral artery compared to the internal carotid artery during the head‐up tilt (Sato et al. 2012). While our study was conducted under baseline supine conditions, we cannot dismiss the possibility that autoregulation of the posterior and anterior cerebrovascular beds may behave differently than the reported MCA values in the OA group.

Despite differences in rate of regulation between groups, rate of regulation is not a standalone index of dynamic cerebral autoregulation. Currently, there is a lack of metric consistency across studies assessing cerebral autoregulation. Numerous variables used to describe cerebral autoregulation result in different interpretations of autoregulation and ensuing implications of such findings. A critical study by Tzeng et al. (2012) assessed the interpretations of cerebral autoregulation by collecting data from different autoregulation challenges and varying follow‐up analytical methods. Their findings highlight a crucial point of consideration when interpreting cerebral autoregulation studies: conclusions drawn from autoregulation studies require careful interpretation and consideration of metrics used to assess autoregulation. In our study, we relied on a single metric, rate of regulation, to provide an index of autoregulation. We also did not report other important metrics that are useful in understanding dynamic autoregulation when using the sit‐to‐stand task as an autoregulation challenge, including but not limited to, time to nadir, relative changes in mean arterial pressure and MCA blood velocity, time to onset of regulatory response (Labrecque et al. 2017; Favre and Serrador 2019; Labrecque et al. 2019). Future studies should explore and provide reference range for the range of autoregulatory variables and metavariables, interpret findings from different context of data collection (sit‐to‐stand vs. thigh cuff), and acquire responses over the range of vasoreactivity (hypo‐,eu‐, and hypercapnia) in individuals with OA.

Therefore, the current data should not be extrapolated to all cases of OA. Rather, these data point to a compelling need for more investigations into the problem of cerebrovascular impairments in OA.

## Summary

This exploratory study is the first to provide preliminary analyses on cerebrovascular control in patients with knee OA in the absence of known cardiovascular disease. While based on a convenience sample of patients, this study provides evidence for sub‐clinical compromise of both peripheral vascular and cerebrovascular health outcomes, placing them at risk for cerebrovascular dysfunction and potential future cerebrovascular events. These observations exist independent of the effects of known cardiovascular/cerebrovascular dysfunction contributing factors. Future larger‐scale studies are required to replicate these findings and contextualize them in clinical settings where severity and type of OA, inflammatory biomarkers, and comorbid risk factors are assessed in tandem with cerebrovascular control.

## Conflict of Interest

The authors have no competing interests to disclose.
